# Mapping an avirulence gene in the sunflower parasitic weed *Orobanche cumana* and characterization of host selection based on virulence alleles

**DOI:** 10.1186/s12870-024-05855-2

**Published:** 2024-11-29

**Authors:** Álvaro Calderón-González, Belén Fernández-Melero, Lidia del Moral, Stéphane Muños, Leonardo Velasco, Begoña Pérez-Vich

**Affiliations:** 1 Instituto de Agricultura Sostenible (IAS-CSIC), Alameda del Obispo s/n, Córdoba, 14004 Spain; 2 Laboratoire des Interactions Plantes Microbes-Environnement (LIPME), Université de Toulouse, CNRS, INRAE, Castanet-Tolosan, France; 3 Present address: Laboratoire des Interactions Plantes Microbes-Environnement (LIPME), Université de Toulouse, CNRS, INRAE, Castanet-Tolosan, France

**Keywords:** Avirulence genes, Genetic mapping, Gene-for-gene interaction, *Orobanche cumana*, Parasitic plants

## Abstract

**Background:**

Sunflower broomrape (*Orobanche cumana* Wallr.) is a holoparasitic plant that jeopardizes sunflower production in most areas of Europe and Asia. Recently, populations with increased virulence, classified as race G_GV_, have been identified in Southern Spain’s Guadalquivir Valley gene pool. These populations overcome resistance genes in hybrids resistant to the predominant race F_GV_. This study aimed to (i) determine the inheritance and map the avirulence trait segregating in a cross between *O. cumana* individuals from populations EK23 (F_GV_) and IN201 (G_GV_), and (ii) characterize the host effect on the IN201 parental population allelic diversity.

**Results:**

A segregating population consisting of 144 F_2:3_ families was evaluated for virulence using a differential sunflower genotype (Hybrid 1, resistant to race F_GV_ and susceptible to race G_GV_) and genotyped with SNP markers. The ratio of avirulent to virulent F_2:3_ families was not significantly different to 1:3 (χ2 = 0.93; *P* = 0.34), indicating monogenic control of the avirulence/virulence trait. The *Avr*_*G−GV*_ locus was mapped on the upper end of *O. cumana* chromosome 2, 9.2 cM distal from the SNP markers OS04791 and OS02805. Secretome analysis in the *Avr*_*G−GV*_ region revealed a cysteine-rich CAP superfamily- and a glucan 1,3-beta-glucosidase family 3-encoding genes as possible candidates for *Avr*_*G−GV*_. SNP allelic analysis on the IN201 population parasitizing a highly susceptible genotype or the differential genotype Hybrid 1 showed that (i) IN201 structure was shaped towards virulent alleles at SNP loci linked to *Avr*_*G−GV*_ (ii) there were significant allelic frequency differences associated with the host genotype at *Avr*_*G−GV*_–linked loci.

**Conclusions:**

This study mapped for the first time an avirulence gene in parasitic plants using a classical genetic approach, confirmed a gene-for-gene model in the *O.cumana* –sunflower system, and showed the implication of this single avirulence gene in determining the structure of broomrape populations subjected to selection pressure posed by a resistant genotype. The results will contribute to a better understanding of the interaction between crops and weedy parasitic plants, and to effectively manage evolution of virulence by sustainable control strategies based on host genetic resistance.

**Supplementary Information:**

The online version contains supplementary material available at 10.1186/s12870-024-05855-2.

## Background

Sunflower broomrape (*Orobanche cumana* Wallr.) is a holoparasitic plant with a very narrow range of hosts. In the wild, it has been observed on a few species of the Compositae, mainly on *Artemisia* spp., whereas in cultivated fields this species only parasitizes on sunflowers [1]. The species is also characterized by the presence of physiological races, i.e., populations with defined virulence patterns [2]. Both aspects differentiate *O. cumana* from other related *Orobanche* spp. and *Phelipanche* spp. parasitizing on agricultural crops, characterized by broader ranges of crop hosts and the absence of well-defined physiological races [3, 4].

Another critical difference between *O. cumana* and other related species of agricultural importance is the genetic control of crop-host resistance. Whereas host resistance in most parasitic systems in which *Orobanche*/*Phelipanche* spp. are involved is under polygenic control (horizontal resistance), resistance to broomrape in sunflower is in most cases vertical i.e., complete, race-specific and controlled by single dominant genes [1]. Thus, the *Or1* to *Or5* genes conferring resistance, respectively, to *O. cumana* races A to E [5], the *HaOr7* to race F [6], and the *Or*_*Deb2*_ and *Or*_*Anom1*_ to race G [7, 8] have been reported.

Vertical resistance is mainly governed by gene-for-gene interactions between resistance genes in the host and the corresponding avirulence genes in the parasite [9]. In the parasitic system involving *O. cumana* and sunflower, this interaction has been not only evaluated from the perspective of the resistance in the host but also through changes of virulence observed in the parasite. Antonova & Ter Borg [10] found that the virulence of *O. cumana* race D against genotypes carrying the *Or3* resistance gene providing resistance to race C was caused by the absence of extracellular excretion of peroxidases from the apex of broomrape radicles, which eluded the formation of lignin layers barriers in sunflower roots. Later, Rodríguez-Ojeda et al. [11] used a classical genetic approach to study the inheritance of avirulence/virulence in populations derived from the cross between *O. cumana* plants of races E and F, using as differential genotype the P-1380 line carrying the resistance gene *Or5* (resistant to race E and susceptible to race F). They concluded that race E avirulence and race F virulence on P-1380 were allelic, and controlled by a single gene named *Avr*_*Or5*_, with avirulence being dominant, as expected for the gene-for-gene interaction.

In Spain, where *O. cumana* is not found in the wild but only as weedy forms in sunflower crops [12], two broomrape gene pools corresponding to separate introductions have been identified [13]. They were distant genetically and geographically, with one found in the central part of the country (Cuenca province) and another in the south (Guadalquivir Valley). Within each pool, very low internal variability was observed, probably due to a founder effect. In the Guadalquivir Valley gene pool, race F became predominant in the middle 1990s and continued without apparent changes in virulence till 2014, when spots of populations attacking race-F-resistant hybrids were detected, being accordingly classified as race G [14]. Greater genetic diversity in these new race G populations compared to conventional race-F populations from the same area was reported [14]. Also, it was concluded as a preliminary hypothesis that the newly detected virulence was caused by recombination of avirulence genes from the two Spanish gene pools of *O. cumana*, which was later confirmed by Fernández-Melero et al. [15] in a similar analysis conducted in the Cuenca gene pool.

Avirulence genes have been studied in plant pathogen organisms such as fungi, bacteria, oomycetes, and viruses [16]. In parasitic plant-crop interactions there are very few studies, focused on identifying protein effectors in *Cuscuta reflexa* (within its interaction with tomato) and *Striga gesnerioides* (parasitizing cowpea) [17, 18]. In the present study, we used a genetic approach to characterize the novel virulence observed in the race-G populations of *O. cumana* of the Guadalquivir Valley. This approach is currently possible thanks to basic genetic research in *O. cumana* carried out in the last decade [11, 19, 20] and the development of genetic and genomic tools for this parasitic weed such as SSR and SNP markers, a genetic linkage map [21, 22] and a genome sequence [23]. Thus, the objectives of this study were to: (i) determine the genetic control of race G virulence through the development of a bi-parental segregating population and its phenotypic evaluation on a sunflower differential host, (ii) map of the avirulence gene(s), (iii) study of avirulence candidates based on the predicted secretome in the genomic region containing the putative avirulence gene, and (iv) analyse population allelic frequencies of markers associated with the avirulence gene in a race-G population showing large intrapopulation genetic diversity.

## Methods

### Plant materials

EK23 is an *O. cumana* race-F population representative of the classical and uniform gene pool of the Guadalquivir Valley in Southern Spain [13]. It was collected in Córdoba in 1995 [11]. IN201 is a sunflower broomrape population collected in Ronda (Málaga, Spain) in 2012 that overcomes resistance provided by race-F resistant hybrids cultivated in the Guadalquivir Valley area and classified accordingly as race G. Following the nomenclature proposed by Martín-Sanz et al. [14], the race for EK23 will be named as F_GV_ and the race for IN201 will be named as G_GV_. Seeds of both *O. cumana* populations are maintained at the Institute for Sustainable Agriculture in Córdoba, Spain. Formal identification of both EK23 and IN201 populations as belonging to the species *O. cumana* has been conducted by Dr. L. Velasco based on morphological characteristics. Additionally, EK23 has been unequivocally identified as a member of the distinct *O. cumana* gene pool of the Guadalquivir Valley in Southern Spain [21], and has been used for the development of the reference genome sequence of the species [23]. Voucher specimens representative of the EK23 or IN201 weedy *O. cumana* populations have not been deposited in a publicly available herbarium, although plant freeze dried material is available from the corresponding author on reasonable request. Nevertheless, a total of thirty-four voucher specimens of *O. cumana* weedy forms representative of populations parasitizing sunflower and also collected in the Guadalquivir Valley area in Southern Spain are deposited in the Herbarium of the University of Córdoba (COA) [12], with some of these populations such as CO196, SE194, and SE296 (voucher specimens deposition numbers COA 28287, COA 28293 and COA 28292, respectively) used in reported virulence studies on sunflower [24].

Six sunflower commercial hybrids resistant to race F_GV_ were used for the characterization of the virulence of the broomrape population IN201. All the hybrids used had been identified as race F_GV_ resistant by the Spanish Plant Variety Registration Office and were active in the Spanish market at the time of the study. The names of the hybrids and the producing seed companies are not provided to not interfere with the commercial interests of the companies, as we did not include hybrids from all companies present in the Spanish market. One of these race-F_GV_ hybrids, named Hybrid Race F-1 in Table 1 and abbreviated as Hybrid 1 in the manuscript, was used as a differential genotype between broomrape populations EK23 and IN201 in the genetic study. The use of a commercial hybrid, for which the genetic profile in relation to resistance genes was unknown to the authors, was mandatory because of the lack of any inbred line resistant to EK23 and susceptible to IN201 that could be used as a differential line. A hybrid resistant to race E but susceptible to race F and the inbred line P96 were also included in the evaluation. P96 is resistant to race F_GV_ [25] and has been reported to be also resistant to race G_GV_ but susceptible to other populations of race G from Eastern Europe [14]. B117 is a confectionery population very susceptible to all *O. cumana* populations evaluated so far [14]. It was used as a susceptible control and for the multiplication of the F_1_ and F_2_ populations.

**Table 1:** Number of emerged shoots per sunflower plant of *Orobanche Cumana* population IN201 against a set of commercial hybrids resistant to race F_GV_ cultivated in Spain in 2017, a race-E resistant hybrid used as susceptible control, and the inbred line P96, resistant to races F_GV_ and G_GV_. Data are given as mean ± standard error

Hybrid/Line	*N*. emerged shoots
Hybrid Race F-1	2.90 ± 0.67
Hybrid Race F-2	0.20 ± 0.20
Hybrid Race F-3	0.00 ± 0.00
Hybrid Race F-4	1.56 ± 0.75
Hybrid Race F-5	0.70 ± 0.42
Hybrid Race F-6	1.10 ± 0.50
P96	0.00 ± 0.00
Hybrid Race E-1	58.70 ± 7.02

### Inheritance study

Flowers of EK23 plants were emasculated before opening and pollinated with fresh pollen from flowers of IN201 plants, following the procedures described by Rodríguez-Ojeda et al. [19]. F_1_ and F_2_ plants were grown on the susceptible line B117, bagged for self-fertilization as described in Rodríguez-Ojeda et al. [19], and harvested individually. The genetic study was based on the study of the virulence reaction of 144 F_2:3_ families (F_3_ seeds from 144 individual F_2_ plants) on the differential genotype Hybrid 1. Additionally, the F_2:3_ families were tested on the susceptible control B117 to check the viability of the seeds. If no parasitization was observed on B117, the F_3_ family was discarded. Each F_2:3_ family was evaluated for virulence reaction on three plants of B117 and six plants of Hybrid 1 in the spring-summer season of 2018. Emerged broomrape shoots were counted on each sunflower plant at maturity. The phenotypic evaluation of F_2:3_ families allowed the F_2_ genotypes to be classified into those possessing race-G virulence (emerged broomrape shoots were observed on plants of B117 and Hybrid 1) and those not possessing race-G virulence (emerged broomrape shoots appeared on plants of B117 but not on plants of Hybrid 1). The virulence of F_1_ genotypes was evaluated in the same environment following the same methodology. Seventeen F_2_ genotypes whose F_3_ phenotypic score was not unequivocal, because they showed a low number of emerged broomrape plants in the susceptible control, were evaluated again following the same procedures. To determine the number of genes involved in the inheritance of the virulence reaction, the goodness of fit between expected and observed phenotypic ratios of F_2_ genotypes was assessed with the chi-square test.

### Inoculation and plant cultivation

Soil inoculation and plant cultivation followed the procedures described by Rodríguez-Ojeda et al. [19]. In short, small pots 7 × 7 × 7 cm were filled with a mixture of sand and peat and 50 mg of broomrape seeds. The mixture was shaken in a plastic bag to distribute broomrape seeds uniformly and put back in the pot. Sunflower seeds were germinated in moistened filter paper at 25ºC in the dark for 48 h. After this time, germinated seeds were planted in the pots, which were maintained in a growth chamber at 25ºC / 20ºC (day/night) with a 16 h photoperiod for eight weeks. The plants were then transplanted into 6-L pots containing a soil mixture of sand, silt, and peat in a proportion 2:1:1. The pots were maintained either in a greenhouse (parents of the crosses and F_2_ generation), growth chamber at the abovementioned conditions (F_1_ generation to produce F_2_ seeds) or open-air conditions in the spring-summer period (F_1_ and F_3_ generations for evaluation of the virulence reaction).

### Tissue collection and DNA extraction

Apical young shoot tissue from the parental populations EK23 and IN201, F_1_ plants, and F_3_ plants from each F_2_ genotype growing on B117 was cut just before flowering. In the case of F_3_ plants tissue was collected from 24 to 30 individual plants from each of the 144 F_2:3_ families and then bulked to recreate the F_2_ genotype. The tissue was maintained in a cooler bag with ice packs during the time of tissue collection and after it was frozen at -80 ºC. It was later lyophilized and ground in a laboratory ball mill. DNA was extracted following the procedure described by Pineda-Martos et al. [13].

### Genetic diversity analysis of parental populations EK23 and IN201 (collected on B117)

An initial diversity analysis was conducted on IN201 to determine its intra-population variability and relatedness with Spanish populations of the Guadalquivir Valley and the Cuenca gene pools. Three populations from each gene pool previously characterized by Rodríguez-Ojeda et al. [19] and Pineda-Martos et al. [13] were used as controls: EK12, CO02, and SE10 from the Guadalquivir Valley, and EKA1, CU05, and CU08 from the Cuenca gene pools. The tissue of individual plants from the *O. cumana* populations EK23 and IN201 was collected on sunflower B117 plants and the DNA extracted as described above. DNA samples from the control populations were those used in the studies described in Pineda-Martos et al. [13] and Calderón-González et al. [21].

The diversity study was conducted using the *O. cumana* 192 SNP set developed by Coque et al. [26] and mapped by Calderón-González et al. [21] (named with SNcum- prefix). These SNPs were distributed across the 19 *O. cumana* linkage groups (LG) [21]. SNcum- markers were genotyped using competitive allele-specific PCR assays based on KASP™ technology at LGC genomics (Teddington, Middlesex, UK) in individuals of the EK23 (*n* = 10), IN201 (*n* = 40), CO02 (*n* = 12), CU05 (*n* = 11), CU08 (*n* = 12), SE10 (*n* = 12), EK12 (*n* = 10) and EKA1 (*n* = 8) populations. For IN201, a higher number of individuals was used because higher intrapopulation diversity was expected, according to the study of Martín-Sanz et al. [14]. After the removal of SNPs that failed or that had more than 10% of missing data, the final SNP set used for the diversity analyses consisted of 152 SNcum- SNPs (Table S1).

For the diversity analyses, the following descriptive parameters were used: percentage of polymorphic loci, allele frequency, Shannon’s information index (I), observed heterozygosity (Ho), Nei’s expected heterozygosity (He), and Nei’s unbiased expected heterozygosity (uHe). A principal coordinate analysis (PCoA) was run using the codominant genetic distance matrix generated from the SNP marker set. GenAlEx 6.503 [27] was used for these analyses.

### Genetic mapping

The set of 192 *O. cumana* SNcum-SNP markers was used for genotyping the parental lines EK23 and IN201 and the mapping population. Genotyping was carried out using KASP™ technology as described above. After an initial genetic linkage analysis in which the LG containing the avirulence locus was identified, 36 additional SNP markers from the same LG with OS prefix mapped in Calderón-González et al. [21] and developed by Coque et al. [26] were also genotyped in the parents and the mapping population. Context sequence for these OS-SNPs is detailed in Table S2. The segregation of alleles at the SNP marker loci was checked against the expected ratios for codominant (1:2:1) markers using a chi-square test. Genetic linkage maps were constructed with MAPMAKER/EXP 3.0b [28] using genotyping data from the SNP polymorphic markers. Map distances in cM were converted from recombination fractions using the Kosambi mapping function. Two-point analysis was used to identify LG with an initial minimum LOD score of 3 and a maximum distance of 30 cM. The identified LG remained consistent with LOD scores ranging from 3 to 10. Three-point and multi-point analyses were used to determine the order and interval distances between the markers in each LG. Linkage maps were drawn using MapChart 2.1 software [29].

The avirulence trait was mapped as a Mendelian locus, since the inheritance study revealed that it was controlled by one single gene, named as *Avr*_*G−GV*_ (detailed in the Results section). Accordingly, the genotypes for the *Avr*_*G−GV*_ gene in each F_2_ plant were inferred from the avirulence/virulence phenotypes in their corresponding F_2:3_ families, as detailed in Table S3. F_2_ plants were classified into *Avr*_*G−GV*_*Avr*_*G−GV*_ (homozygous for the avirulent allele) when no emerged broomrape shoots on the differential hybrid were observed after inoculation with their F_3_ seeds. Conversely, F_2_ plants were classified as *Avr*_*G−GV*_*avr*_*G−GV*_ or *avr*_*G−GV*_*avr*_*G−GV*_ when the differential genotype Hybrid 1 showed some degree of parasitization after inoculation with their F_3_ seeds. Unequivocal separation of *Avr*_*G−GV*_*avr*_*G−GV*_*and avr*_*G−GV*_*avr*_*G−GV*_ genotypes was not feasible by phenotypic evaluation, since in both cases their F_2:3_ progeny shows parasitization on Hybrid 1 (Table S3). *Avr*_*G−GV*_ mapping was carried out as indicated for SNP markers, using a LOD threshold of 15 as the linkage criterium. Additionally, the significance of each marker’s association with the phenotypic trait was determined by one-way analysis of variance (ANOVA) using the statistical package SPSS Statistics v 29.0 (IBM, Armonk, NY, USA), with marker genotypes as classes.

### Candidate gene analysis based on the *Avr*_*G−GV*_-associated secretome

The protein-coding genes located in the genomic region where the *Avr*_*G−GV*_ gene was mapped were extracted from the annotated *O. cumana* chromosome-level genome sequence (OcIN23-20190821) [23, 30] and processed for secretome identification. For the prediction of the secretory proteins, the secretory signal peptide was uncovered using SignalP-6.0 (https://services.healthtech.dtu.dk/services/SignalP-6.0) [31]. Since SignalP cannot detect transmembrane proteins, we used DeepTMHMM (https://dtu.biolib.com/DeepTMHMM) [32] for this purpose. Final signal peptide protein selection was achieved using SignalP-TMHMM prediction. We also used OutCyte 1.0 (http://www.outcyte.com) [33] to predict proteins that are secreted via ‘non-conventional’ signal peptide-independent mechanisms. Finally, the organelle-based localization predictions were performed using DeepLoc2.0 (https://services.healthtech.dtu.dk/services/DeepLoc-2.0/) [34] and BUSCA (https://busca.biocomp.unibo.it/) [35]. Only the consensus hits predicted as secretory and ‘extracellular’ by all methods were considered for further analysis. The secreted protein candidates were classified according to their Gene Ontology terms and their functional description using InterPro (https://www.ebi.ac.uk/interpro/) [36]. Furthermore, they were characterized through BlastP sequence searches on the non-redundant protein sequence library at NCBI [37], the pathogen-host interaction database (PHI-base) (http://phi-blast.phi-base.org/, 4.14 protein sequences) [38], and a race-G *O. cumana* transcriptional profile filtered for predicted secreted proteins during sunflower susceptible and resistant interactions [39].

### Host-dependent genotypic structure of the IN201 population

Allelic frequencies at SNP loci were determined within the IN201 population on plants parasitizing either the susceptible population B117 or the differential Hybrid 1. For that, B117 and Hybrid 1 plants were grown on soil inoculated with seeds of the IN201 population, following the previously described procedures. The plants were grown in the greenhouse in the winter of 2020/21. Apical tissue of 69 individual plants of IN201 parasitizing B117 and 80 individual plants of IN201 parasitizing Hybrid 1 was collected for DNA extraction, as described above. Allelic frequencies and the percentage of individuals within each SNP-genotypic class were determined for the SNP loci located in the LG in which the avirulent gene was mapped, as well as at SNP loci randomly distributed across other LG. In the former case, all the SNP markers with the prefix OS were used [21]. In the latter case, we used a set of 68 SNP with the prefix SNcum- [21] that were polymorphic in the IN201 population based on the genetic diversity study previously conducted on this population (Table S1). SNP genotyping was carried out using KASP™ technology as described above. GenAlEx 6.503 [27] was used for allelic frequency calculations. A comparison of means between allelic frequencies on plants collected on B117 and Hybrid 1 was conducted using paired-*t* tests on arcsin-transformed frequencies [40]. The influence of the host on the distribution of the IN201 individuals within the SNP genotypic classes was tested with Pearson’s chi-square analysis [41] conducted using SPSS Statistics v 29.0 (IBM, Armonk, NY, USA).

## Results

### Virulence of IN201

Population IN201 was virulent against five of the six commercial hybrids with resistance to race F_GV_ used in the study (Table 1). It was also virulent against the race-E resistant hybrid and avirulent against P96. The degree of attack, i.e. the number of emerged broomrape shoots per sunflower plant, was low in all susceptible hybrids with race-F resistance, from 0.20 ± 0.20 (mean ± standard error) to 2.90 ± 0.67, while it was very high in the race-E resistant hybrid (58.70 ± 7.02) (Table 1).

### Genetic diversity analysis of EK23 and IN201 populations

Population EK23 showed zero values for all the intra-population diversity parameters evaluated, as did the three control populations from the Guadalquivir Valley gene pool EK12, CO02, and SE10, and the three control populations from the Cuenca gene pool EKA1, CU05, and CU08 (Table 2). Compared to these populations, IN201 showed considerably higher genetic diversity in all evaluated parameters (Table 2).

**Table 2:** Percentage of polymorphic loci (PL), and mean values for Shannon’s information index (I), observed heterozygosity (Ho), Nei’s expected heterozygosity (he), and Nei’s unbiased expected heterozygosity (uHe) in the parental populations EK23 (race F_GV_) and IN201 (race G_GV_), the three populations from the Guadalquivir Valley gene pool EK12, CO02 and SE10, and the three populations from the Cuenca gene pool EKA1, CU05 and CU08. All populations were collected on B117

	PL (%)	I	Ho	He	uHe
EK23 (*n* = 10)	0.00	0.00	0.00	0.00	0.00
IN201 (*n* = 40)	49.34	0.20	0.04	0.12	0.12
*Guadalquivir Valley gene pool controls*					
EK12 (*n* = 10)	0.00	0.00	0.00	0.00	0.00
CO02 (*n* = 12)	0.00	0.00	0.00	0.00	0.00
SE10 (*n* = 12)	0.00	0.00	0.00	0.00	0.00
*Cuenca gene pool controls*					
EKA1 (*n* = 8)	0.00	0.00	0.00	0.00	0.00
CU05 (*n* = 11)	0.00	0.00	0.00	0.00	0.00
CU08 (*n* = 12)	0.00	0.00	0.00	0.00	0.00

**Fig. 1:**
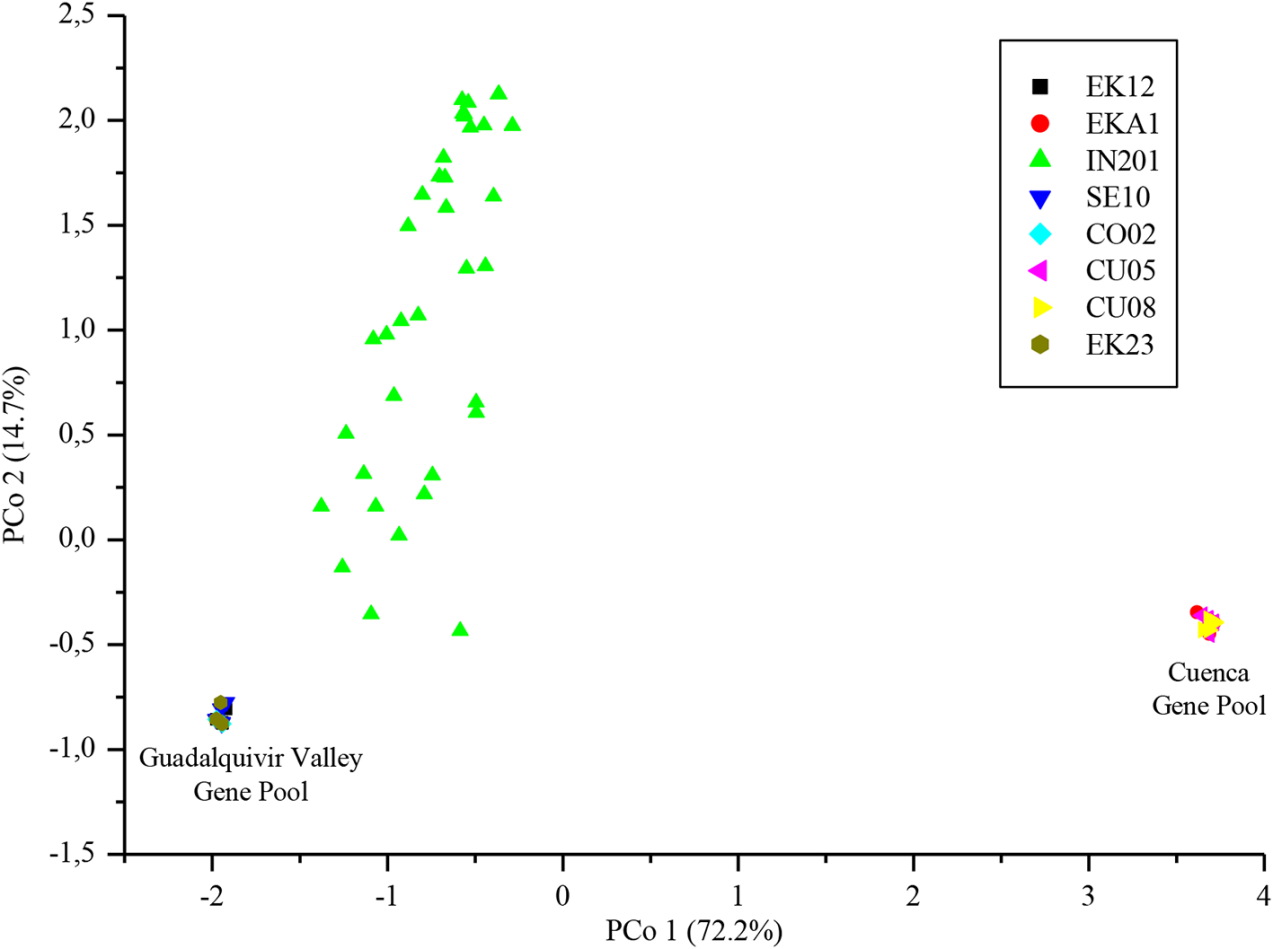
Principal coordinate analysis of the *Orobanche cumana* parental populations EK23 (F_GV_) and IN201 (G_GV_), and six reference populations from the Guadalquivir Valley (GV) (EK12, CO02 and SE10) and from the Cuenca (EKA1 CU05 and CU08) gene pools, all parasitizing sunflower genotype B117

The three first axes from the principal coordinate analysis conducted using the eight populations explained 72.17%, 14.65%, and 2.10% of the total variation, respectively. Figure 1 shows the biplot of the two first axes. All the individuals from the populations of the Guadalquivir Valley gene pool were grouped together. Similarly, all the individuals from the populations of the Cuenca gene pool were also grouped together, in a distant group. Individuals from population IN201 showed a high dispersion and were closer to the Guadalquivir Valley than to the Cuenca gene pool.

### Inheritance study

The race-F_GV_ parent EK23 was avirulent on Hybrid 1 and virulent on the susceptible control B117 (17.13 ± 3.12 shoots per plant given as mean ± standard error). The race-G_GV_ parent IN201 was virulent on both B117 (29.50 ± 2.54) and Hybrid 1 (10.38 ± 2.03). F_1_ plants from the cross between EK23 and IN201 were also virulent on both B117 (22.33 ± 4.39) and Hybrid 1 (2.09 ± 0.53). Taking into account the phenotypic midparent value, the data suggested partial dominance of avirulence over virulence.

All the 144 F_2:3_ families (F_3_ plants from each individual self-pollinated F_2_ plant) were virulent on B117, with an average number of shoots per plant from 1.00 to 60.00. On Hybrid 1, there were 31 F_2:3_ families avirulent and 113 virulent, with an average number of broomrape shoots per plant from 0.20 to 16.33. The ratio of avirulent to virulent families was not significantly different to 1:3 (χ^2^ = 0.93; *P* = 0.34), which indicated monogenic inheritance of the trait. The gene was named as *Avr*_*G−GV*_. Considering only the families that were virulent on Hybrid 1, there was a positive correlation between the number of shoots per plant on B117 and Hybrid 1 (*r* = 0.65; *P* < 0.01), suggesting that part of the differences between families in the number of emerged shoots per plant were caused by differences in germination ability and/or seed vigour.

### Genetic mapping of the *Avr*_*G−GV*_ gene

**Fig. 2:**
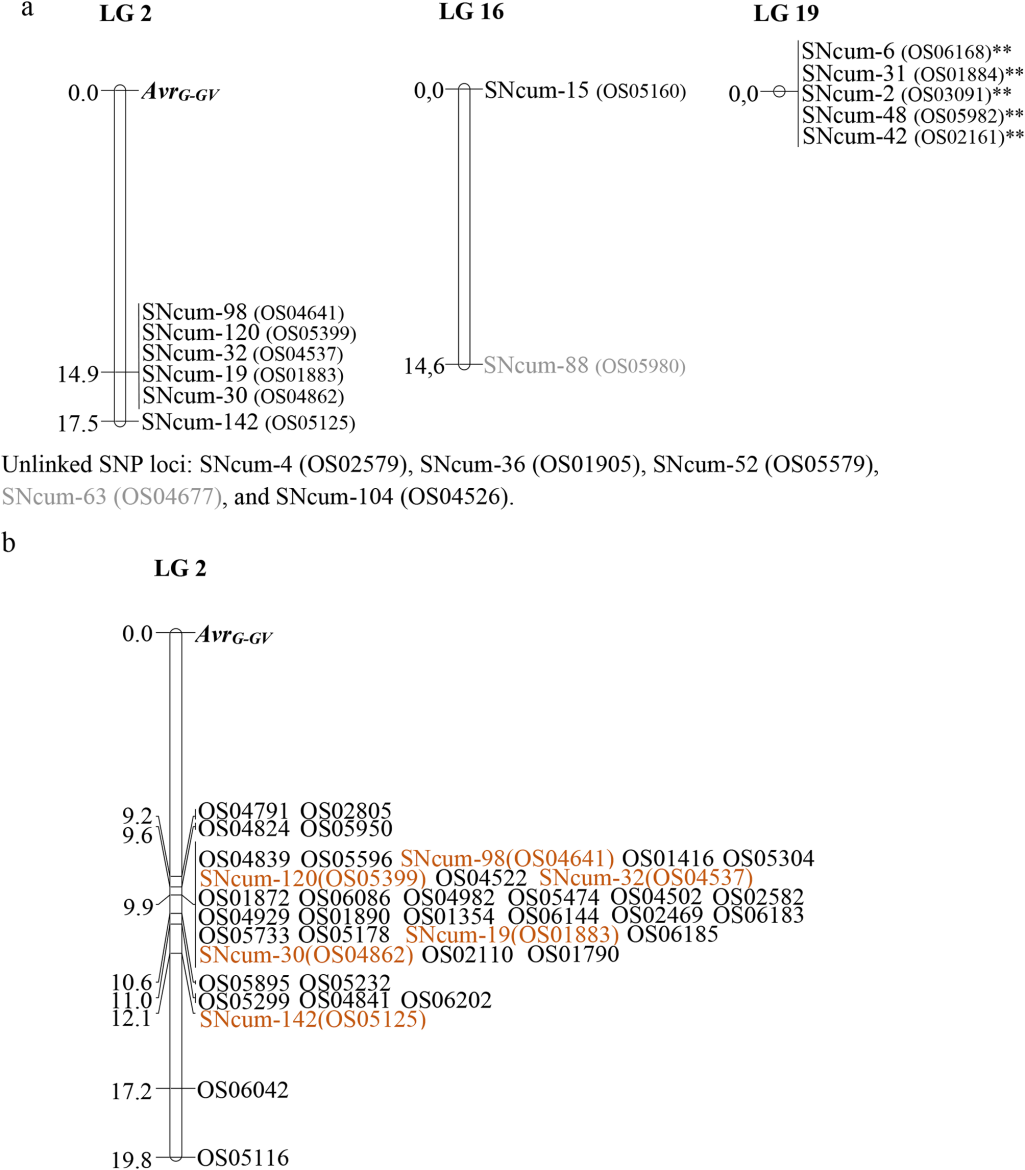
Genetic mapping of the avirulence gene *Avr*_*G−GV*_. SNP marker loci labelled as ** showed distorted segregation at *P* < 0.01. (**a**) Initial map constructed after screening with a 192 SNcum-SNP set distributed across the *O. cumana* map. Black coloured SNP loci were also mapped in Calderón-González et al. [21], (**b**) LG 2 map constructed after screening with an OS-SNP set mapped previously to LG 2

From the 192 *O. cumana* SNPs (SNcum prefix), only 18 were polymorphic and segregated in the EK23 x IN201 mapping population. In 17, polymorphism was codominant. The polymorphic SNP loci were arranged into three LGs with 6, 5 and 2 markers, respectively (Fig. 2a). The remaining five SNP loci were unmapped. The 13 markers arranged in LGs mapped to LG 2 (6 SNPs), LG 19 (5 SNPs), and LG 16 (2 SNPs) of the *O. cumana* map (Fig. 2a). All the marker loci from LG 19 showed distorted segregation at *P* < 0.01 and they were clustered at a 0 cM distance. Also, most of the markers on LG 2 were mapped in a cluster, although distorted segregation was not detected for any of them (Fig. 2a). The locus *Avr*_*G−GV*_ was mapped on the upper end of LG 2 (Fig. 2a).

Of the 36 OS LG2 SNPs, 3 were monomorphic and 33 polymorphic, the latter being used for the construction of the LG 2 final map containing the *Avr*_*G−GV*_ gene (Fig. 2b, Table S4). Overall marker order was coincident with that described in Calderón-González et al. [21], although marker clustering was more pronounced in this study. The *Avr*_*G−GV*_ gene was mapped to the upper end of LG 2, with markers OS04791 and OS02805 9.2 cM away (Fig. 2b). ANOVA analyses indicated significant differences (*P* < 0.001) between the marker class means for the total number of broomrape shoots per plant in the differential Hybrid 1 at all LG 2 markers, while no significant differences were found at markers in the other LG (Table 3), and at unlinked markers. At all LG 2 markers, the virulent allele was that from the IN201 parent (Table 3). It is worth mentioning that the LG 2 avirulent allele, i.e., that present in EK23, was also present in other populations of the Guadalquivir Valley gene pool used in this study, whereas the alternative virulent allele, i.e., that present in IN201, was in all markers coincident with the one found in the Cuenca gene pool. Finally, no significant differences between the marker class means were found at any marker for the total number of broomrapes in the susceptible control line B117 (Table 3).

**Table 3:** Association between SNP mapped marker loci (for markers mapping clustered at 0 cM, only one representative marker per cluster is included) and the total number of broomrapes (per plant) emerged in either the differential hybrid 1 or the susceptible control line B117 determined by variance analysis in the EK23 x IN201 mapping population. Marker closest to the *Avr*_*G−GV*_ gene is highlighted in bold

LG	Marker	Sunflower host	No. individuals within each marker class			Mean ± SD for the number of broomrapes per plant within each marker class^a^			ANOVA	
			A	H	B	A (EK23)	H	B (IN201)	*F*	*P*
2	**OS04791**	**Hybrid 1**	**37**	**60**	**42**	**0.4 ± 1.0**	**3.7 ± 3.1**	**4.7 ± 4.1**	**21.64**	**< 0.001**
		**B117**	**37**	**60**	**42**	**14.9 ± 8.6**	**19.7 ± 13.3**	**18.7 ± 14.1**	**1.76**	**0.18**
2	OS05950	Hybrid 1	37	59	44	0.4 ± 1.0	3.7 ± 3.1	4.6 ± 4.1	20.25	< 0.001
		B117	37	59	44	14.9 ± 8.6	19.9 ± 13.6	18.2 ± 13.9	1.76	0.18
2	OS04839	Hybrid 1	37	62	42	0.4 ± 1.0	3.6 ± 3.1	4.7 ± 4.1	21.55	< 0.001
		B117	37	62	42	14.9 ± 8.6	19.3 ± 13.6	18.8 ± 14.1	1.49	0.23
2	OS5895	Hybrid 1	37	61	45	0.4 ± 1.0	3.3 ± 2.8	4.9 ± 4.1	22.8	< 0.001
		B117	37	61	45	14.9 ± 8.6	18.1 ± 12.7	18.9 ± 13.8	1.22	0.30
2	OS6202	Hybrid 1	37	61	43	0.4 ± 1.0	3.5 ± 2.9	5.0 ± 4.2	23.53	< 0.001
		B117	37	61	43	14.9 ± 8.6	19.5 ± 13.5	19.0 ± 14.0	1.66	0.20
2	SNcum-142	Hybrid 1	40	57	41	0.8 ± 1.8	3.4 ± 2.9	5.1 ± 4.2	19.70	< 0.001
		B117	40	57	41	15.3 ± 8.6	19.4 ± 13.9	19.3 ± 14.1	1.5	0.23
2	OS6042	Hybrid 1	34	58	46	1.0 ± 2.0	3.2 ± 3.0	4.6 ± 4.2	12.22	< 0.001
		B117	34	58	46	15.7 ± 9.0	19.2 ± 13.5	19.1 ± 13.7	0.94	0.39
2	OS5116	Hybrid 1	34	62	41	1.0 ± 2.0	3.5 ± 3.4	4.3 ± 4.0	9.96	< 0.001
		B117	34	62	41	15.5 ± 9.4	19.3 ± 14.6	18.8 ± 11.9	1.09	0.34
16	SNcum-15	Hybrid 1	37	72	30	2.8 ± 3.6	3.5 ± 3.6	2.7 ± 2.9	0.78	0.46
		B117	37	72	30	16.9 ± 12.2	19.1 ± 13.5	18.1 ± 11.5	0.34	0.71
16	SNcum-88	Hybrid 1	36	77	27	2.9 ± 3.5	3.5 ± 3.7	2.3 ± 2.5	1.36	0.26
		B117	36	77	27	15.4 ± 10.3	20.3 ± 13.9	16.0 ± 10.0	2.43	0.09
19	SNcum-42	Hybrid 1	51	65	24	2.7 ± 2.8	3.5 ± 3.8	3.1 ± 3.9	0.72	0.49
		B117	51	65	24	17.1 ± 12.8	19.1 ± 13.1	17.2 ± 12.2	0.39	0.68

^a^Mean number of broomrapes per plant ± standard deviation (SD) are presented in different genotypic classes: A, homozygous with respect to the avirulent allele derived from EK23; B, homozygous with respect to the virulent allele derived from IN201; H, heterozygous

### Candidate virulence gene analysis based on the *Avr*_*G−GV*_ associated secretome

**Fig. 3:**
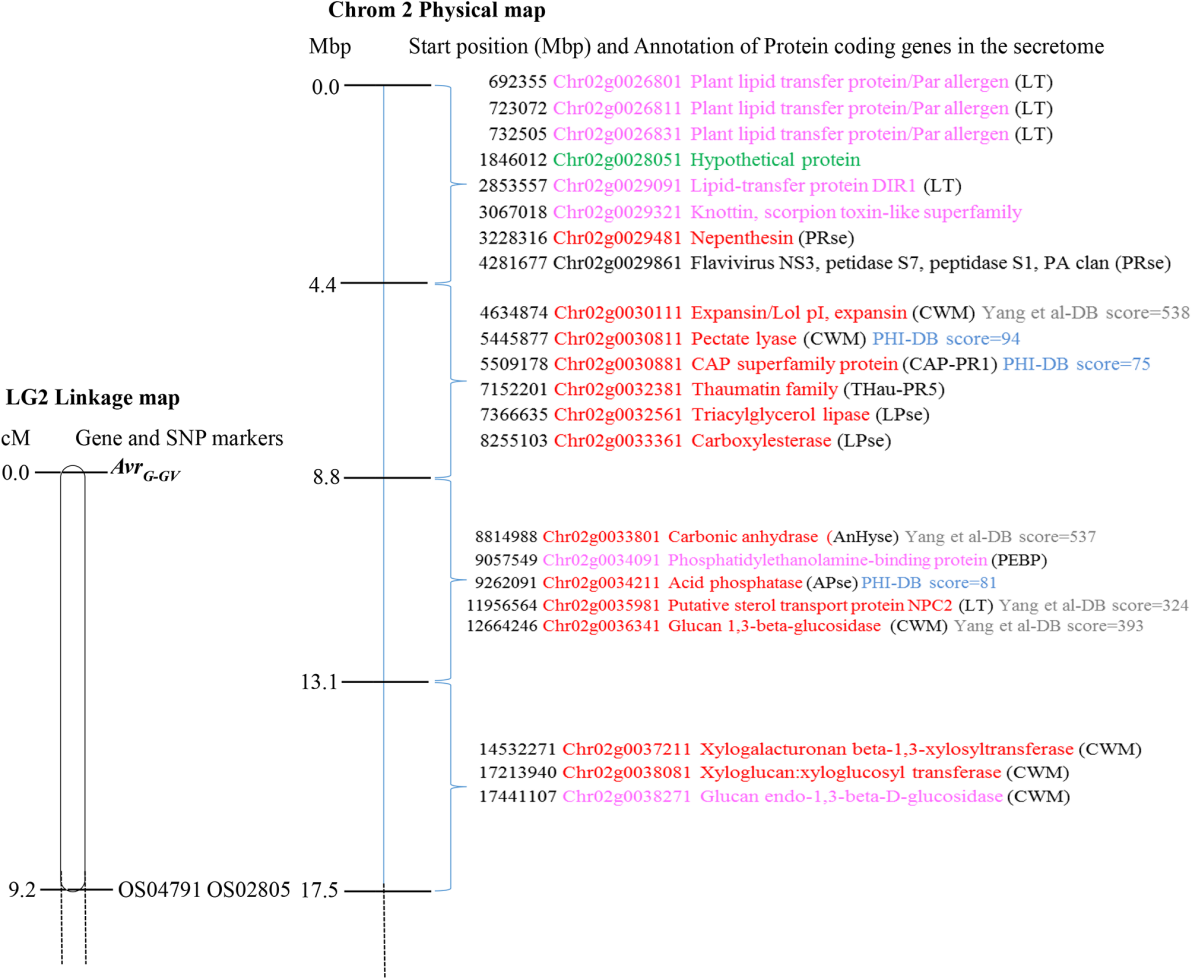
Relative position for *Avr*_*G−GV*_ secretome candidate genes in the physical *O. cumana* map (right) in Mbp compared to the *Avr*_*G−GV*_ linkage map (left) in cM. The functional groups in which the genes are categorised are showed according to the following labels: LT = Lipid transport, CWM = Cell wall modification, PRse = Proteinases, LPse = Lipases, AnHyse = Anhydrase, CAP-PR1 = Cysteine-rich secretory protein with CAP domain (pathogenesis-related proteins of the PR1 family), THau-PR5: Thaumatin (pathogenesis-related group 5 protein), PEBP = PhosphatidylEthanolamine-Binding Protein, and APse = Acid phosphatase. Genes are coloured according to BlastP results as follows: Red_Best hit alignment score > = 200; Pink_from 80 to 200; Green_from 50 to 80; and Black_ No hits. The scores of the bests Blast hits against the PHI-base and the race G *Orobanche cumana* secretory transcriptional profile of Yang et al. [39] are also indicated and labelled in blue and grey, respectively

A total of 1082 protein-coding genes were found in the annotated *O. cumana* genome sequence within the *Avr*_*G−GV*_ region delimited by the closest markers OS04791 and OS02805 (17.47 Mbp from the Chromosome 2 upper end). Twenty-two were secretory proteins predicted to be extracellular (Table S5, Table S6, Fig. 3). Thirteen of these 22 secretory proteins showed high score blast hits (≥ 200) against the NCBI non-redundant protein database. Among them, there were (i) four genes showing very high score BLAST hits (from 324 to 538) against the race G *O. cumana* secretory filtered transcriptional profile [39], and (ii) three genes with medium-score BLAST hits (from 75 to 94) against the PHI-base (Table S5, Fig. 3). Most (77.2%) of the 22 predicted secretory proteins had InterPro functional domains and were categorized into different functional groups, such as genes associated to cell wall modification, involved in lipid transport, proteases and lipases (Table S5 and Fig. 3). There were also five genes encoding proteins with a range of putative functions, including a cysteine-rich secretory protein with CAP domain (pathogenesis-related group 1 protein), a thaumatin (pathogenesis-related group 5 protein), an anhydrase, a phosphatidyl/ethanolamine-binding protein, and an acid phosphatase (Table S5, Fig. 3).

### Host-dependent genotypic structure of the IN201 population

**Fig. 4:**
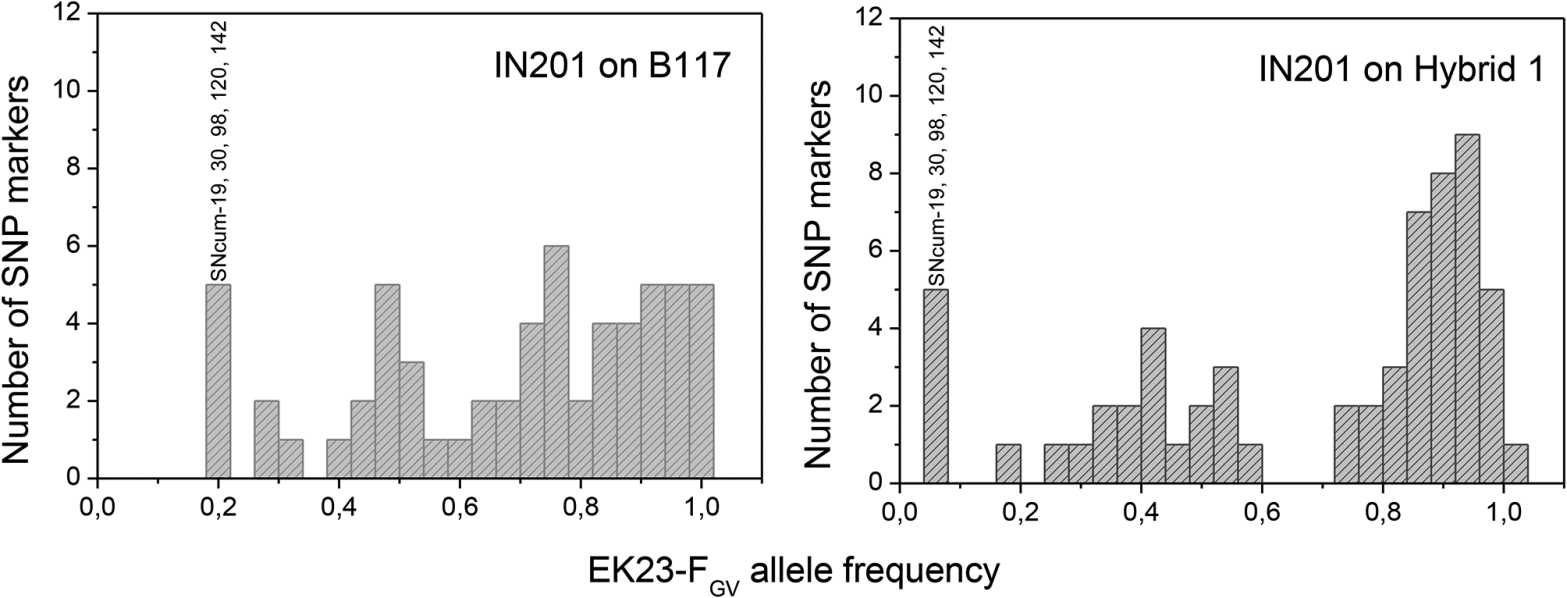
Frequency distribution of the allele from the avirulent race F_GV_ parental population EK23 in IN201 plants parasitizing on sunflower genotypes B117 and Hybrid 1

Sixty-three SNcum SNP markers, distributed across LGs, were polymorphic in the IN201 population. They all contained either the allele found in the Guadalquivir Valley gene pool (populations EK23, EK12, CO02, and SE10), or the allele of the Cuenca gene pool (populations EKA1, CU05, and CU08). The frequency of the EK23-Guadalquivir Valley allele in the SNP markers ranged from 0.20 to 1.00 in plants collected on B117 and from 0.07 to 1.00 in plants collected on Hybrid 1 (Fig. 4). Considering all the LG, no significant differences (*t* = 0.58; *P* > 0.05) in the average frequency of EK23-Guadalquivir Valley alleles were observed between IN201 plants collected on B117 (0.69 ± 0.25) and on Hybrid 1 (0.68 ± 0.30). However, an interesting observation was that the markers with the lowest frequency of EK23-Guadalquivir Valley alleles were all located at LG 2, where the *Avr*_*G−GV*_ locus was mapped, both in the set of plants collected on B117 and in those collected on Hybrid 1. They were SNcum-19, 30, 98, 120, and 142, with frequencies of the EK23-Guadalquivir Valley allele from 0.20 to 0.21 for plants collected on B117 and from 0.07 to 0.08 for plants collected on Hybrid 1 (Fig. 4). Based on this observation, we repeated the comparative analysis of the frequencies of the EK23-Guadalquivir Valley alleles in B117 and Hybrid 1, but this time restricted to the markers located on LG2 that were polymorphic in this population, including both the markers with SNCum- and OS prefix (Table S7). In this case, the average allelic frequency in IN201 plants collected on B117 (0.16) was significantly different (*t* = 16.93; *P* < 0.01) from the average allelic frequency in plants collected on Hybrid 1 (0.05).

**Fig. 5:**
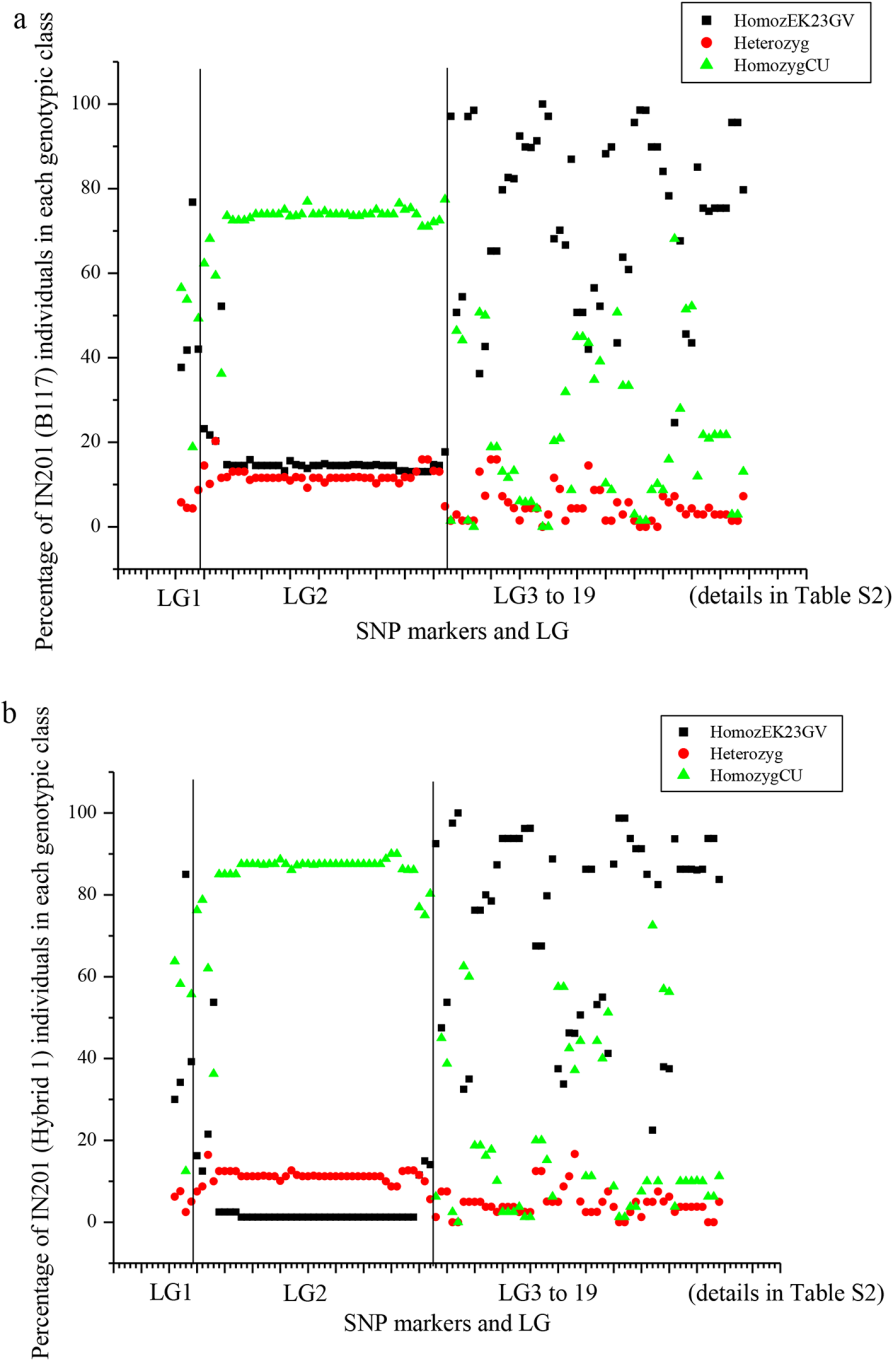
Scatter plot of the percentage of individuals at each SNP genotypic class (homozygous for the EK23 allele, heterozygous, and homozygous for the alternative allele, present in the Cuenca gene pool) in the IN201 parental population (Y-axis) across SNP loci at the different linkage groups of the *Orobanche cumana* map (X-axis), as detailed in Table S7. The X-axis does not reflect the size of LGs, but the number of markers at each LG, which is considerably higher in LG 2 where the *Avr*_*G−GV*_ gene has been mapped (**a**) IN201 collected on B117, (**b**) IN201 collected on Hybrid 1

**Fig. 6:**
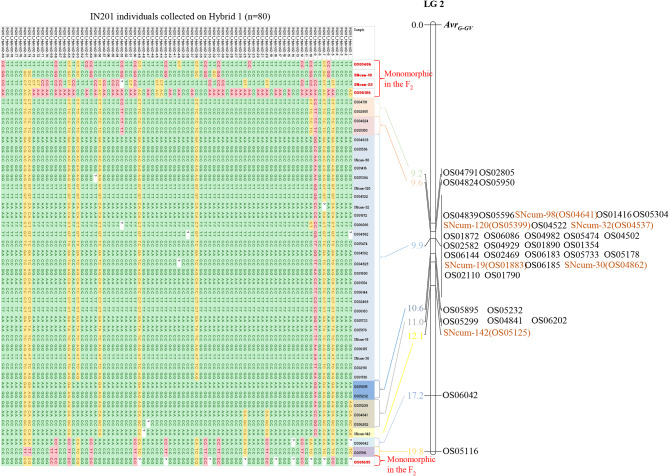
Haplotypes at LG2 SNP loci in individuals of the race G_GV_ parental population IN201 collected on Hybrid 1. The genotypes of IN201 individuals (grown on Hybrid 1) (columns) are represented across the SNPs markers (rows). The genotype homozygous for the EK23-F_GV_ (avirulent) allele is represented in pink, that homozygous for the IN201-alternate allele (virulent) in green, that heterozygous in yellow and missing data in white. As a reference, it is represented on the right of the figure the mapping position of each SNP marker in the LG 2 EK23 x IN201 genetic map. Monomorphic markers not segregating in this mapping population are labelled in red

At the single SNP level, differences in their allelic configuration between IN201 plants collected on B117 and Hybrid 1 were significant (*p* < 0.05) in 36 correlative SNP markers of LG2, one SNP marker from LG8, and one SNP marker from LG16, whereas they were not significant in seven markers at the extremes of LG2 and the remaining 54 markers of the other LGs (Table S7). The low representation of the EK23-F_GV_ allele in LG2 markers, more markedly in plants collected on Hybrid 1, in contrast to the predominance of this allele in the other LGs, is clearly observed in Fig. 5, which shows the genotypic classes at each SNP marker used in this study. Figure 6 details more specifically the haplotypic configuration at LG2 of the 80 IN201 plants collected on the differential genotype Hybrid 1, in which an unequivocal association between the Cuenca gene pool (virulent) haplotype at this LG and virulence on Hybrid 1 can be observed. It is worth mention that this haplotype configuration was not conserved at the upper- and lower-most regions of LG 2 (Fig. 6).

## Discussion

The results of the present study revealed that the difference in virulence between broomrape populations EK23, of race F_GV_, and IN201, of the new race G_GV_ with higher virulence, is caused by the single gene *Avr*_*G−GV*_. This confirms previous results based on phenotypic segregation data obtained by Rodríguez-Ojeda et al. [11], who also found monogenic segregation in a cross between plants from *O. cumana* populations of races E_GV_ and F_GV_. Single genes associated with pathogenic virulence have been described in different gene-for-gene interactions involving pathogens other than parasitic plants, such as bacteria, fungi or nematodes [16].

The analysis of the F_1_ generation from the EK23 (race F_GV_) x IN201 (race G_GV_) cross revealed partial dominance of race F_GV_ avirulence over race G_GV_ virulence. Previously mentioned research by Rodríguez-Ojeda et al. [11] reported that race E_GV_ avirulent alleles were dominant over F_GV_ virulent alleles. It is important to note that in that study, *O. cumana* F_1_ plants from the race E_GV_ x race F_GV_ cross were tested against the P-1380 inbred line, homozygous for the *Or5* resistance gene. In the present study, the F_1_ generation was tested against hybrid (heterozygous) material, since a sunflower race G_GV_ differential inbred line was not available. This may explain the observed partial dominance rather than the complete dominance often expected in gene-for-gene interactions. There is very little information regarding the dominance relationships of avirulence/virulence alleles in *O. cumana* and, in general, in weedy parasitic plants, making it difficult to contextualize this hypothesis within existing research. However, it is worth mentioning that partial dominance of avirulence over virulence, as observed here, has also been documented in other gene-for-gene pathosystems not involving parasitic plants. For example, a similar behavior for specific isolates has been observed in genetic studies conducted on *Bremia lactucae* (downy mildew) infecting lettuce (*Lactuca sativa*) [42], which was explained by the authors by the modification of the expression of certain alleles depending on the genetic background of host and pathogen.

The existence of a new race G_GV_ population of sunflower broomrape that overcame the resistance provided by the race-F resistant hybrids cultivated in the Guadalquivir Valley was reported for the first time by Martín-Sanz et al. [14]. The authors found that the increased virulence was associated with larger intra-population diversity. They hypothesized that the genetic recombination of virulence alleles caused it after the introduction of plants of the Cuenca gene pool (race F_CU_) into the Guadalquivir Valley followed by spontaneous crosses between plants of both gene pools (races F_GV_ and F_CU_). In this study, we could not evaluate F_CU_ populations because there were no viable seeds available from populations that can be definitively considered pure representatives of the F_CU_ gene pool. This is because all populations collected in recent years either included individuals from the Guadalquivir Valley gene pool or displayed varying levels of virulence [15]. Nonetheless, the results of the present research fully support the hypothesis by Martín-Sanz et al. [14], since the alleles of the Cuenca (F_CU_) gene pool have been unequivocally associated with the increased virulence of the Guadalquivir Valley race G_GV_ population IN201. It is also worth mentioning that in the cross between the *O. cumana* populations EK23 (race F_GV_) and IN201 (putatively race F_GV_ + F_CU_), only the virulence locus of the F_CU_ gene pool was expected to segregate if the hypothesis was correct, which fits with the observed results of our study. The process of genetic recombination between *O. cumana* populations from the Guadalquivir Valley and Cuenca gene pools has also been documented recently in populations collected in Cuenca [15]. Genetic recombination of virulence alleles has already been described as a mechanism that can give rise to novel virulent phenotypes in fungi, oomycetes, and nematodes [43]. In the case of sunflower as a host, increased virulence of downy mildew (*Plasmopara halstedii*) in France was reported to be caused by admixture and hybridization between populations of different races [44].

The IN201 (race G_GV_) degree of attack was higher in the race-E-resistant hybrid than in the race-F-resistant hybrids. This has also been reported previously for other broomrape populations also classified as race G_GV_ [14]. Two main factors can explain this. First, broomrape populations can be heterogeneous in virulence [24]. Thus, broomrape population IN201 may contain race-G seeds that can parasitize race-F-resistant hybrids and race-F seeds that will parasitize race-E-resistant hybrids but not race-F-resistant hybrids. Second, sunflower hybrids with resistance to race F may carry minor genes contributing to broomrape resistance in addition to major genes. Race F in Spain appeared in the middle 1990s, only a few years after race E was identified [45], and it was predominant till 2014, when race G was first detected [14]. Accordingly, breeding intensity for resistance to broomrape has been higher for race-F-resistant hybrids. Since we used commercial hybrids, it is not possible to know the details of their genetic background.

The *Avr*_*G−GV*_ locus has been mapped on the upper part LG 2 of the *O. cumana* genetic map, 9.2 cM distal from the SNP markers OS04791 and OS02805. These two markers are located at 18.3 cM from the upper end of the LG 2 linkage map, and at a 17.5 Mbp physical position in Chromosome 2 of the *O. cumana* chromosome-level genome sequence. It must be considered that the *Avr*_*G−GV*_ gene has been mapped through its phenotypic score in a segregating bi-parental populations by virtue of its ability to infect the race F_GV_ resistant differential genotype Hybrid 1. To circumvent phenotyping difficulties inherent to *O. cumana* parasitism, we used (i) F_2:3_ families as the testing generation to be able to detect avirulent genotypes, and (ii) simultaneous F_2:3_ family evaluation in a host susceptible genotype (B117), in addition to the differential host (Hybrid 1), to confirm the parasitic ability of the seeds. Nevertheless, the dominant score of *Avr*_*G−GV*_, coupled with the possibility of phenotyping misclassification that cannot be entirely dismissed, suggests the necessity for a more precise mapping of the *Avr*_*G−GV*_ locus. This can be achieved, for example, through the utilization of recombinant inbred populations, which are presently in the developmental stages and offer the advantages of being immortal and showing a phenotypic co-dominant score.

Marker polymorphisms and order of the current LG 2 genetic map were highly congruous with that of the map described in Calderón-González et al. [21], although a more pronounced clustering of markers on LG 2 was observed in this study. Marker clustering has been described in genetic maps in other plant species as reflecting low levels of recombination [46], which have been associated with either centromeric regions [47] or non-centromeric heterochromatin and other repeat-rich regions [48]. Alternatively, low recombination rates could be caused by chromosomal re-arrangements or regions of low sequence homology that are specific to the parental combination used to construct the mapping family [49]. Also, the lack of recombination around genes with evolutionary significance has been reported [50, 51]. Understanding the rationale behind marker clustering in LG 2 will require further research and will benefit from mapping additional *Avr* genes and conducting further genomic research on this parasitic species.

Proper race identification is one of the main bottlenecks in *O. cumana* research. Despite eight *O. cumana* races (named with letters from A to H) have been reported to date in several countries [52], the terminology used for race identification is confusing because the same race nomenclature is used in different geographical areas for diverse parasite populations [1]. For example, Martín-Sanz et al. [14] reported differences in virulence between populations classified as race G from Spain and from Eastern Europe countries. Molecular population diversity studies with overall genome scans have not been useful for race identification in *O. cumana* [13, 53–55]. In parasitic systems governed by gene-for-gene interactions, this can be theoretically explained because host selection could act upon just a single gene that determines virulence, while the rest of the genome is shaped by other evolutionary forces [56]. Given that the effectiveness of major resistance genes is tightly dependent on the respective *Avr* gene, molecular markers associated with *Avr* genes could be used as a diagnostic tool for providing DNA-based information of the virulence spectrum of a pathogen population, as it has been demonstrated, for example, in the fungal pathogen *Magnaporthe oryzae* that causes rice blast disease [57]. In this sense, molecular markers reported in this study linked to *Avr*_*G−GV*_ represent a starting point for developing early, reliable, and sensitive diagnostic tools for race G_GV_ identification. Nevertheless, a more precise mapping of the *Avr*_*G−GV*_ locus, using new markers (i.e. based on the *O. cumana* genome) to test for new polymorphisms, and testing and validating the markers are needed before a molecular race diagnosis could be implemented.

The nature of avirulence genes in parasitic plants remains poorly understood. Recent studies in gene-for-gene interactions between crops and parasitic plants evidence that parasitic plant-host interactions might be governed by similar rules as in other plant pathogen-host interactions [58]. In this way, it has been demonstrated that parasitic plants can produce effector proteins that suppress host plant immunity, e.g., the *Striga gesnerioides* race SG4z parasitizing cowpea (*Vigna unguiculata*) cultivar B301 [18]. Also, pathogen-associated molecular patterns (PAMPs) that are recognized by host receptors leading to defense responses have been recently described, such as the PAMP GRP (glycine-rich protein) from *Cuscuta reflexa*, which acts as a binding ligand for the tomato (*Solanum lycopersicum*) receptor protein *CuRe1* triggering plant defense responses [17].

In this study, the combined approach of mapping the avirulence gene and the analysis of the secretome in the *Avr*_*G−GV*_ region resulted in twenty-two putative avirulence candidates. A strong candidate was a putative cysteine-rich CAP superfamily protein (OcIN23r2_Chr02g0030881), described previously as important for the suppression of the host´s immune response in phytopathogenic fungi and nematodes [59]. Moreover, these proteins have been described as effectors interacting indirectly with cell-surface pattern recognition receptors (PRRs) in gene-for-gene recognition events [60–62]. Also, it is worth mentioning that OcIN23r2_Chr02g0030881 showed sequence similarity in the pathogen-host interaction database to two CAP-domain-containing genes from the pathogen species *Candida albicans* and *Fusarium graminearum* (Table S5), which, when disrupted, caused reduced virulence in their corresponding hosts [63, 64]. On the other hand, there were four other genes showing high homology with race-G *O. cumana* predicted secreted proteins described by Yang et al. [39] (Fig. 3). Among them, a glucan 1,3-beta-glucosidase family 3 (OcIN23r2_Chr02g0036341) was also considered as a good candidate, since its homolog (TR252940|c0_g1_i1, Glycosyl hydrolase family 3) showed a differential expression in sunflower race G susceptible vs. race G resistant reactions [39] and a glycosyl hydrolase family 3 was reported as a candidate virulence factor in the parasitic plant *Striga hermonthica* [65].

Different life-history traits can influence gene-pool dynamics and population structure in the weedy species of *Orobanche* [56]. In agroecosystems in which resistant genotypes are broadly cultivated, parasitic weed populations might undergo changes in allelic frequencies due to selective pressures posed by the cultivation of resistant cultivars [56, 65]. Vertical resistance has been widely used in sunflower because it facilitates considerably hybrid sunflower breeding [1]. Nevertheless, its efficacy diminishes over time due to the significant selection pressure exerted on the *Avr* gene through successive use of resistant genotypes. This pressure favors mutations within the gene, particularly those rendering their product unrecognizable by resistance genes, swiftly gaining predominance within the pathogen population [66]. This has been demonstrated in the present study in the race-G_GV_-virulent population IN201, where the population structure is shaped towards the virulent allele at the LG 2 genomic region containing the *Avr*_*G−GV*_ gene (Fig. 4). Additionally, we observed a direct impact of host genetics in filtering the IN201 population, which showed significant enrichment of virulent alleles at the LG 2 *Avr*_*G−GV*_ gene when grown on the resistant genotype Hybrid 1 as compared with the susceptible one B117. All these observations illustrate (i) how the selective pressures may primarily act upon the allelic frequencies of markers associated with an avirulence gene, being host-induced selection one of the most important selective forces in *O. cumana* in the context of agricultural ecosystems and (ii) the importance of the gene-for-gene interaction in driving *O. cumana* population dynamics. Breeding strategies aimed at reducing this strong selection pressure have been proposed for a more durable resistance, mainly combining major resistance genes and QTL, preferably with complementary modes of action [67, 68].

Although molecular mechanisms responsible for plant parasitism have been extensively studied over the last decade [66, 69], research on avirulence genes in gene-for-gene systems within parasitic plants remains limited. This study reports for the first time a map position for an avirulence gene within parasitic plants, provides new evidence that the sunflower-*O. cumana* parasitic system follows a gene-for-gene model, highlights the role of the *Avr* gene in shaping broomrape population structures under the selection pressure of resistance genes, and proposes a handful of candidate genes whose actual role in virulence will need to be further validated. In conclusion, this research represents a significant contribution to understanding virulence in *O. cumana* and it lays the groundwork for *Avr* gene map-based cloning strategies and for race classification of *O. cumana* populations based on the presence of virulent alleles at this *Avr* gene.

## Electronic supplementary material

Below is the link to the electronic supplementary material.

Supplementary Material 1

Supplementary Material 2

Supplementary Material 3

Supplementary Material 4

Supplementary Material 5

Supplementary Material 6

Supplementary Material 7

## Data Availability

SNcum-SNP context sequences are reported in Calderón-González et al. [21]. LG2 OS-SNP context sequences are detailed in Table S2. Amino acid sequences for the AvrG-GV candidate genes are indicated in Table S6. Any other data that support the findings of this study are available from the corresponding author on reasonable request.
