# Supplementary material for: Mapping an avirulence gene in the sunflower parasitic weed Orobanche cumana and characterization of host selection based on virulence alleles
Source: BMC Plant Biol. 2024 Nov 29;24:1147. doi: 10.1186/s12870-024-05855-2 (PMC11606015; doi:10.1186/s12870-024-05855-2)
Supplement: Supplementary file 3 — Supplementary Material 3 [file 12870_2024_5855_MOESM3_ESM.pdf]

Table S3. Detailed expected genotypes and phenotypes in the F_3 families from each self-pollinated broomrape F_2 plant obtained from the cross between parents EK-23 and IN201.

F_2 plant genotype (for the avirulence/virulence gene)	F_2 plant expected phenotype on Hybrid 1	Expected genotype(s) (for the avirulence/virulence gene) for each F_3 family from individual self-pollinated F_2 plants	F_3 family phenotype on Hybrid 1
Avr/Avr (homozygous for EK-23 avirulent allele)	Avirulent- No broomrape. (Non-parasitizing F_2 individuals can not be evaluated at the F_2 level)	Non-segregating *Avr/Avr*	Avirulent- No broomrape
Avr/avr (heterozygous)	Partial dominance of avirulence vs virulence- Emerged broomrape shoots	Segregating *Avr/Avr* + *Avr/avr*+ *avr/avr*	Mixture of genotypes *Avr/Avr* (avirulent) + *Avr/avr* (partial dominance) + *avr/avr* (virulent)- Emerged broomrape shoots
avr/avr (homozygous for IN201 virulent allele)	Virulent- Emerged broomrape shoots	Non-segregating *avr/avr*	Virulent- Emerged broomrape shoots

Following this scheme, the calculation of the segregation ratios in the genetic study is as follows. First, the F_2 plant genotype (from individual self-pollinated F_2 plants) is inferred from its corresponding F_3 family evaluation as follows:

- No broomrape on Hybrid 1 in the F_3. F_2 genotype: *Avr/Avr*.

- Broomrape shoots on Hybrid 1in the F_3. F_2 genotype: *Avr/avr* or *avr/avr*.

Second, the calculation of the segregation ratios is based on the proportion of inferred F_2 genotypes *Avr/Avr* (no broomrape shoots in their F_3 progeny) versus *Avr/avr* + *avr/avr* (broomrape shoots in their F_3 progeny).
